# Inhibitory effect of *trans*-ferulic acid on proliferation and migration of human lung cancer cells accompanied with increased endogenous reactive oxygen species and β-catenin instability

**DOI:** 10.1186/s13020-016-0116-7

**Published:** 2016-10-01

**Authors:** Yao Fong, Chia-Chun Tang, Huei-Ting Hu, Hsin-Yu Fang, Bing-Hung Chen, Chang-Yi Wu, Shyng-Shiou Yuan, Hui-Min David Wang, Yen-Chun Chen, Yen-Ni Teng, Chien-Chih Chiu

**Affiliations:** 1 Department of Thoracic Surgery, Chi-Mei Medical Center, Tainan, 710 Taiwan; 2 Division of Chest, Ten Chan General Hospital, Chung-Li, 320 Taiwan, ROC; 3 Department of Biotechnology, Kaohsiung Medical University, Kaohsiung, 807 Taiwan; 4 Department of Food Nutrition, Chung-Hwa University of Medical Technology, Tainan, 701 Taiwan; 5 Department of Biological Sciences, National Sun Yat-sen University, Kaohsiung, 804 Taiwan; 6 Translational Research Center, Kaohsiung Medical University Hospital, Kaohsiung, 807 Taiwan; 7 Graduate Institute of Biomedical Engineering, National Chung Hsing University, Taichung, 402 Taiwan; 8 Department of Biological Sciences and Technology, National University of Tainan, Tainan, 700 Taiwan; 9 Research Center for Environment Medicine, Kaohsiung Medical University, Kaohsiung, 807 Taiwan; 10 The Institute of Biomedical Sciences, National Sun Yat-Sen University, Kaohsiung, 804 Taiwan

## Abstract

**Background:**

*Trans*-ferulic (FA) acid exhibits antioxidant effects in vitro. However, the underlying mechanism of *trans*-FA activity in cellular physiology, especially cancer physiology, remains largely unknown. This study investigated the cellular physiological effects of *trans*-FA on the H1299 human lung cancer cell line.

**Methods:**

The 2,2-diphenyl-1-picrylhydrazyl assay was used to determine free radical scavenging capability. Assessment of intracellular reactive oxygen species (ROS) was evaluated using oxidized 2ʹ,7ʹ-dichlorofluorescin diacetate and dihydroethidium staining. Trypan blue exclusion, colony formation, and anchorage-independent growth assays were used to determine cellular proliferation. Annexin V staining assay was used to assess cellular apoptosis by flow cytometry. Wound healing and Boyden’s well assays were used to detect the migration and invasion of cells. Gelatin zymography was used to detect matrix metalloproteinase (MMP-2 and MMP-9) activity. Western blotting was used to detect expression levels of various signaling pathway proteins.

**Results:**

DPPH assay results indicated that *trans*-FA exerted potent antioxidant effects. However, *trans*-FA increased intracellular ROS levels, including hydrogen peroxide and superoxide anion, in H1299 cells. *Trans*-FA treatment inhibited cellular proliferation and induced moderate apoptotic cell death at the highest concentration used (0.6 mM). Furthermore, *trans*-FA moderately inhibited the migration of H1299 cells at the concentrations of 0.3 and 0.6 mM and attenuated MMP-2 and MMP-9 activity. *Trans*-FA caused the phosphorylation of β-catenin, resulting in proteasomal degradation of β-catenin. Conversely, *trans*-FA treatment increased the expression of pro-apoptotic factor Bax and decreased the expression of pro-survival factor survivin.

**Conclusion:**

Various concentrations (0.06–0.6 mM) of *trans*-FA exert both anti-proliferation and anti-migration effects in the human lung cancer cell line H1299.

**Electronic supplementary material:**

The online version of this article (doi:10.1186/s13020-016-0116-7) contains supplementary material, which is available to authorized users.

## Background

In 2013, the mortality rates for male and female lung cancer patients in the United States were 28 and 26 %, respectively [1]. At present, chemotherapy is the primary treatment for lung cancer [2–4], with both carboplatin and cisplatin commonly used as chemotherapy drugs [5, 6]. However, the combination of cisplatin or vinblastine with irradiation increases the level of unexpected toxicity in the body [4]. Pemetrexed (Alimta^®^), a next-generation antifolate drug, is used for treating malignant pleural mesothelioma and non-small cell lung cancer (NSCLC) but can induce scleroderma-like induration of the lower extremities [7]. It is therefore necessary to develop alternative treatment strategies for selective inhibition of lung cancer cells growth.

The carcinogenic process may be driven by mutations, leading to alterations in phenotypes, genetics, and epigenetics. The induction of oxidative stress in various cancer cells such as human pancreatic and colon adenocarcinoma cancer cell lines [8] was shown to inhibit expression of β-catenin and the matrix metalloproteinases MMP-2 and MMP-9 in colitis-associated colon carcinoma [9], induce Bax expression in urothelial cell carcinoma [10], induce apoptosis by blocking the AMPK-mTOR-survivin pathway [11], and inhibit the anchorage-independent growth (AIG) of transformed cells [12]. Chemopreventive treatment is moderate or non-cytotoxic to normal cells, but significantly inhibits cancer cell growth or metastasis. Accumulating evidence shows that anti-oxidative compounds isolated from plants exert potentially chemopreventive effects. Among these compounds are carotenoids, curcumin, and hesperidin [13, 14]. Recently, anticancer compounds derived from plants, including goniothalamin and feruloyl-l-arabinose, were shown to inhibit the growth and metastasis of human lung cancer cells [15, 16]. Additionally, moscatilin, isolated from the orchid *Dendrobrium loddigesii*, inhibited metastasis of both human breast and lung cancer cells [17, 18].

Ferulic acid (FA) is an aromatic compound, abundant in plant cell walls [19, 20]. Both isomers of FA, *cis*-FA and *trans*-FA, show a potent ability to remove reactive oxygen species (ROS) and inhibit lipid peroxidation [20, 21]. Unlike *cis*-FA, *trans*-FA is abundant in plant cells and easily isolated from various plants, thus significantly reducing the cost of its preparation [22].

*Trans*-FA ameliorated ionizing radiation-induced inflammation and glycerol-induced nephrotoxicity [23, 24] and modulated fluoride-induced oxidative hepatotoxicity in male Wistar rats [25]. Water-soluble *trans*-FA sugar esters protected normal rat erythrocytes against peroxyl radical 2,2ʹ-azobis-2-amidinopropane dihydrochloride (AAPH)-induced oxidative damage [26] and exhibited protective capacity against oxidative damage caused by diabetes [27, 28]. Furthermore, *trans*-FA exhibited antiproliferative effects in colon cancer cells [29, 30], increased the radiosensitivity of cervical cancer cells [31], and exerted protective effects against chemical-induced DNA strand breaks [32, 33]. However, the effects of *trans*-FA in lung cancer have not been reported to date, and its biological mechanism remains unknown.

The study investigated the cellular and physiological effects of *trans*-FA in human lung cancer cells.

## Methods

### Chemicals

*Trans*-FA (4-hydroxy-3-methoxycinnamic acid) was purchased from Sigma-Aldrich Chemicals (#128708, St. Louis, MO, USA). *Trans*-FA was freshly dissolved in 0.01 % dimethyl sulfoxide (DMSO) and aliquoted before use.

### Cell culture

The human non-small cell lung cancer (NSCLC) cell line H1299 and lung fibroblast cells HEL-299 were obtained from the American Type Culture Collection (ATCC, Manassas, VA, USA). Cells were maintained in DMEM:F-12 medium (1:1 ratio) supplemented with 8 % fetal bovine serum, 2 mM glutamine, 100 units/mL penicillin, and 100 µg/mL streptomycin (Gibco BRL, Gaithersburg, MD, USA) at 37 °C in a humidified atmosphere of 5 % CO_2_ [4].

### DPPH radical-scavenging activity assay

The anti-oxidant activities of *trans*-FA were measured based on the scavenging activity of 2,2-diphenyl-1-picrylhydrazyl (DPPH) (#D9132, Sigma-Aldrich) free radical [34, 35]. Briefly, vitamin C standards and various *trans*-FA concentrations were freshly prepared and diluted in methanol. Methanol (as a blank control; 10 µM) or *trans*-FA solution was added to 90 µL DPPH solution to yield a final *trans*-FA concentration of 0.15 mg/mL in a 96-well microplate. The mixture was incubated at 25 °C and protected from light. After incubation, solution absorbance was measured at 492 nm using a Multiskan Ascent 354 microplate reader (Thermo Fisher Scientific, Rockford, IL, USA). DPPH radical scavenging activity was calculated as follows:

$${\text{DPPH radical scavenging activity }}\left( \% \right) \, = \, \left( { 1- A_{0} /A_{ 1} } \right) \, \times { 1}00$$

where *A*_0_ and *A*_1_ are the absorbances of the control and *trans*-FA solutions, respectively. Each experiment was repeated three times and found to be reproducible within experimental error margins.

### Cell viability and proliferation assay

Briefly, 5 × 10^4^ cells were seeded into wells of a 12-well plate and treated with phosphate-buffered saline (PBS) (Sigma-Aldrich) as control or concentrations of *trans*-FA (0.03–0.6 mM) for 24 or 48 h. After incubation, cell viability and proliferation were analyzed by trypan blue assay and an automated cell counter (Countess™) according to the manufacturer’s instructions (Invitrogen, Carlsbad, CA, USA).

### Colony formation assay

Fifty cells were seeded into wells of a 6-well plate and, after 24 h of incubation, were treated with different concentrations of *trans*-FA (0.03–0.6 mM). After incubation for 11 days, cell colonies were glutaraldehyde-fixed and stained with crystal violet (0.1 % w/v) for 10 min. Colony diameter was determined using Image-Pro v3.0 software (Media Cybernetics, Silver Spring, MD, USA).

### Anchorage-independent growth (AIG) assay

The assay procedure was performed as described in our previous work, with minor modifications [36]. Briefly, 1 × 10^3^ cells were mixed with 0.75 % low melting agarose (MDBio Inc, Taipei, Taiwan). The mixtures were placed on a solidified layer of 1.5 % agarose with medium in a 12-well plate. After incubation for 13 days, cells in the upper layer were fixed with 1 % glutaraldehyde and stained with 0.1 % w/v Giemsa (Merck, Darmstadt, Germany). Colony diameter was determined using Image-Pro v3.0 software.

### Cell cycle analysis

Cell cycle distribution was assessed using propidium iodide (PI; Sigma-Aldrich) staining described previously [37]. Briefly, 1 × 10^5^ cells were treated with PBS (as vehicle control) or various *trans*-FA concentrations (0.03–0.6 mM) for 48 h. Next, cells were detached using 0.05 % trypsin (Biological Industries, Kibbutz Beit Haemek, Israel) for 5 min, harvested, and washed with PBS. Cells were then fixed with 75 % ethanol overnight. After centrifugation (Microfuge^®^ 16, Beckman Coulter Life Sciences, Taipei, Taiwan) at 664×*g* for 15 min at 4 °C, the resulting supernatants were decanted. Cell pellets were stained with 10 µg/mL PI and 10 µg/mL RNase A (Sigma-Aldrich) in PBS buffer for 30 min at 37 °C in the dark. The samples were assayed using a FACScan flow cytometer (Becton–Dickinson, Mansfield, MA, USA) and the results were analyzed using FlowJo v7.5.5 software (Tree Star Inc., San Carlos, CA).

### Assessment of apoptosis

Apoptotic cell death was assessed by annexin V and PI double staining (Pharmingen, San Diego, CA, USA) according to our previous paper [15]. Briefly, 1 × 10^6^ cells were seeded into a 100-mm petri dish and treated with vehicle or *trans*-FA at doses of 0–0.6 mM for 48 h. Next, cells were inoculated with 10 µg/mL of annexin V-fluorescein isothiocyanate (FITC) and 20 µg/mL of PI and analyzed using a FACScan flow cytometer FlowJo v7.5.5 software.

### Detection of endogenous ROS

Changes in endogenous ROS levels were assessed using the fluorescent indicators 2′,7′-dichlorofluorescin diacetate (DCFDA, Sigma-Aldrich) for hydrogen peroxide (H_2_O_2_) and dihydroethidium (DHE) (Invitrogen) for superoxide anion (O_2_^−^). A total of 1 × 10^5^ H1299 cells were seeded into wells of a 6-well culture plate and treated with various concentrations (from 0.03 to 0.15 mM) of *trans*-FA for 24 h. Next, cells were harvested and stained with 100 nM DCFDA or 1 µM DHE for 30 min at 37 °C in PBS and then washed twice with PBS. Fluorescence was measured by flow cytometry.

### Western blot assay

Western blot assays were performed as described previously [38]. Briefly, cells were harvested and lysed, lysates were centrifuged, and protein concentrations of cell pellets were determined. Next, 40 mg quantities of protein lysate were resolved by 10 % SDS–polyacrylamide gel electrophoresis and electro-transferred to polyvinylidene difluoride membranes. The membranes were blocked with 5 % nonfat milk and incubated with the following primary antibodies: Thr^41^/Ser^45^ phosphorylated β-catenin (#2377-S, Epitomics, CA, USA), β-catenin (#sc-7963, Santa Cruz Biotech., CA, USA), Bax (#ab32503, Abcam Inc., Cambridge, MA, USA), survivin (#614701, Biolegend, CA, USA), and β-actin (#AM1021b, Abgent, San Diego, CA, USA), followed by appropriate secondary antibodies (anti-Mouse, #074-1806; anti-Rabbit, #074-1506, KPL, Gaithersburg, MD, USA). The WesternBright™ ECL kit (Advansta, CA, USA) was used for signal detection.

### Wound-healing assay

Quantities of 3 × 10^5^ H1299 cells were seeded in wells of a 12-well plate, treated with PBS (as vehicle control) or *trans*-FA (0.03–0.6 mM), and grown to 100 % confluence. Culture monolayers were scratched using a pipette tip to create a clean 1-mm-wide wound area. After further incubation for 16 h, the wound gaps were photographed and analyzed using TScratch software (CSE Lab, Zurich, Switzerland) [39].

### Transwell invasion assay

Invasion assays were performed as described in our previous work, with minor modifications [40], using 8 µm-pore Transwell^®^ chambers (Greiner Bio-One, Frickenhausen, Germany). Control and various concentrations (from 0.03 to 0.6 mM) of *trans*-FA treated cells were cultured in triplicate at 5 × 10^4^ cells/well in the upper inserts of 24-well Transwell^®^ culture plates. Next, cells were fixed for 5 min and stained with 0.1 % w/v Giemsa. The cells which had invaded the lower inserts were counted by arbitrarily selecting five fields from each well. The experiments were repeated three times.

### Gelatin zymography

MMP-2 and -9 activity was assessed by gelatin zymography as previously described [41], with minor modification. Briefly, 3 × 10^5^ H1299 cells were seeded into wells of 12-well plates and cultured with various concentrations of *trans*-FA for 24 h, after which aliquots of culture medium were harvested for gelatin zymography analysis. Samples were prepared in standard SDS-PAGE loading buffer containing 0.01 % SDS without β-mercaptoethanol and were not boiled before use. Next, the samples were subjected to electrophoresis (150 V for 3 h) on 10 % SDS–polyacrylamide gels containing 1 % gelatin. After electrophoresis, the gels were thoroughly washed with distilled water containing 2.5 % Triton X-100 on a gyratory shaker for 30 min at room temperature. Gels were incubated in 100 mL reaction buffer (40 mM Tris–HCl, pH 8.0; 10 mM CaCl_2_, and 0.02 % NaN_3_) at 37 °C overnight, followed by staining with Coomassie brilliant blue R-250 (Bio basic Inc., Markham, Ontario, Canada) and de-staining with methanol-acetic acid–water (50/75/875, v/v/v). The gelatinase activities of MMP-2 and -9 were determined by analyzing signal intensity using Gel Pro v.4.0 software (Media Cybernetics, Silver Spring, MD, USA).

### Statistical analysis

All data are mean ± SD from at least three experiments, with three replicates per experiment. The significance of the differences was analyzed using one-way analysis of variance (ANOVA) and SigmaPlot v12.0 software (Systat Software Inc., San Jose, CA, USA). *P* values less than 0.05 were considered statistically significant.

## Results

### Radical scavenging activity of *trans*-FA

**Fig. 1:**
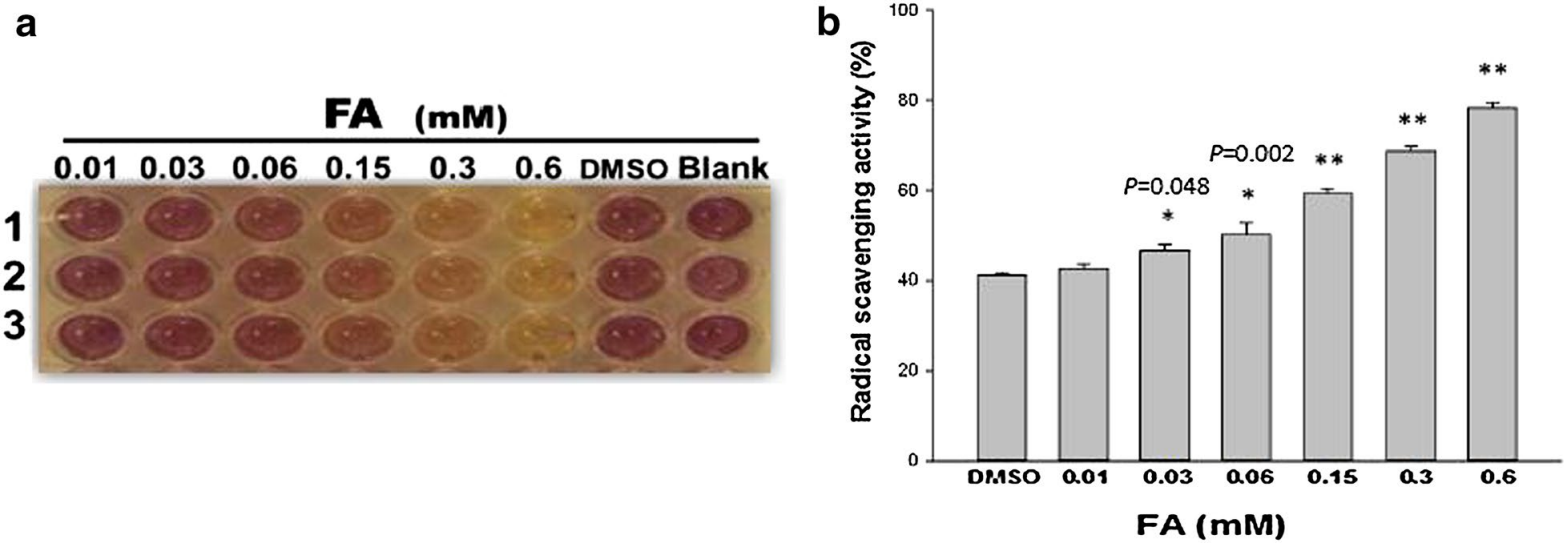
DPPH radical-scavenging capacity of *trans*-FA. **a** *Trans*-FA was tested in an antioxidant assay by measuring the DPPH radical-scavenging activity. The indicated concentrations of *trans*-FA were incubated with DPPH respectively as described in the “Methods” section. **b** Quantificative analysis. The radical-scavenging capacity of *trans*-FA at indicated concentrations was quantified as the percentage decrease in absorbance at 492 nm against the DMSO control. *P < 0.05 and **P < 0.001, respectively

The DPPH assay was used to assess the radical scavenging activity of *trans*-FA. Antioxidant activity was calculated by evaluating the capacity of *trans*-FA to scavenge DPPH. As shown in Fig. 1, *trans*-FA treatment produced significant radical-scavenging activity compared with the DMSO control. Vitamin C was serially diluted in methanol (500–7.8 µM) and used in triplicate as a positive control (Additional file 1).

### The short-term effect of *trans*-FA on proliferation of NSCLC cells

**Fig. 2:**
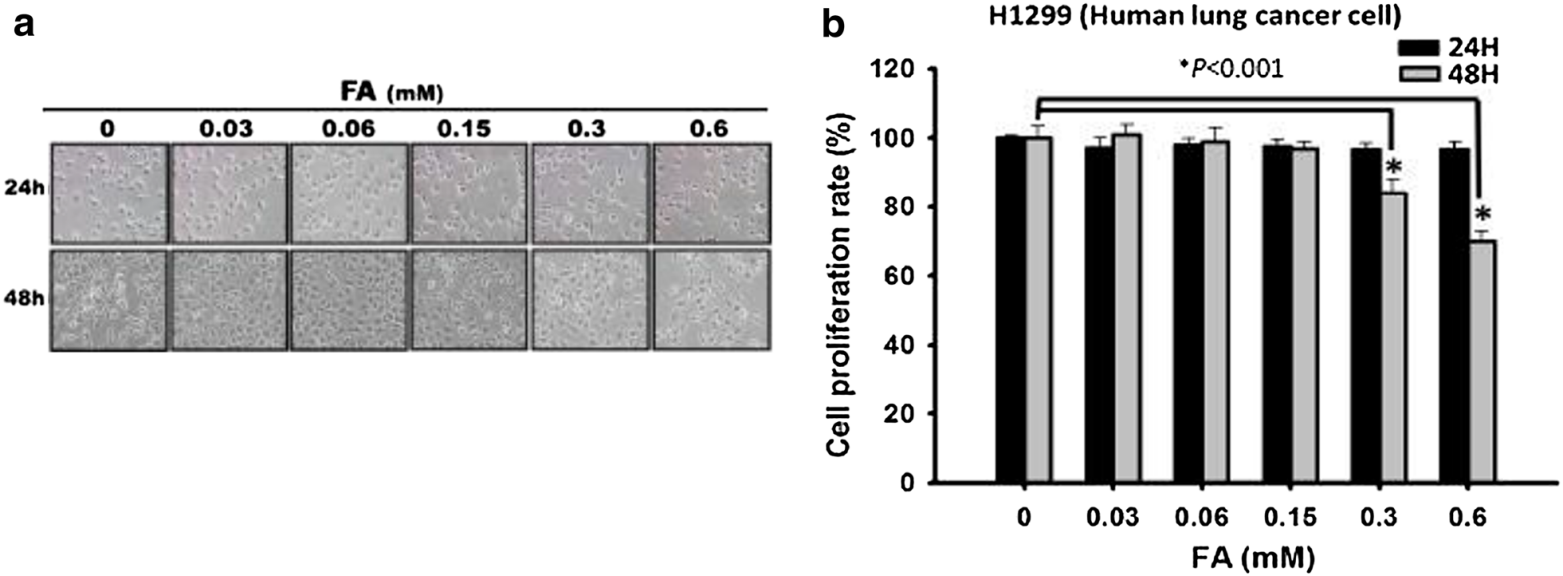
Comparison of cell morphology and proliferation between of control and *trans*-FA-treated of NSCLC cells. **a** H1299 cells were treated with PBS as vehicle control or the indicated doses of *trans*-FA for 24 and 48 h, respectively. And then, morphology was examined and photographed with a light microscope. (phase contrast, X200). **b** 5 × 10^4^ H1299 cells were seeded onto a 12-well plate, and cells were treated with indicated doses of *trans*-FA for 24 and 48 h, respectively. Cell viability was determined using the trypan blue exclusion assay described in “Methods” section. *P < 0.001 against vehicle control

H1299 cells were treated with PBS (as vehicle control) or different concentrations of *trans*-FA for 24 or 48 h before gross morphological changes were examined by light microscopy to determine the effect of *trans*-FA on cell growth (Fig. 2a). Cells exhibited no significant change in morphology compared with the vehicle control. Next, measurement of cell survival was performed by trypan blue dye exclusion assay. Low doses (>0.15 mM) of *trans*-FA exerted no significant cytotoxic effect, but moderate cytotoxicity was observed with 0.3 and 0.6 mM treatment for 48 h (Fig. 2b).

### The long-term effect of *trans*-FA on colony formation and AIG assay in NSCLC cells

**Fig. 3:**
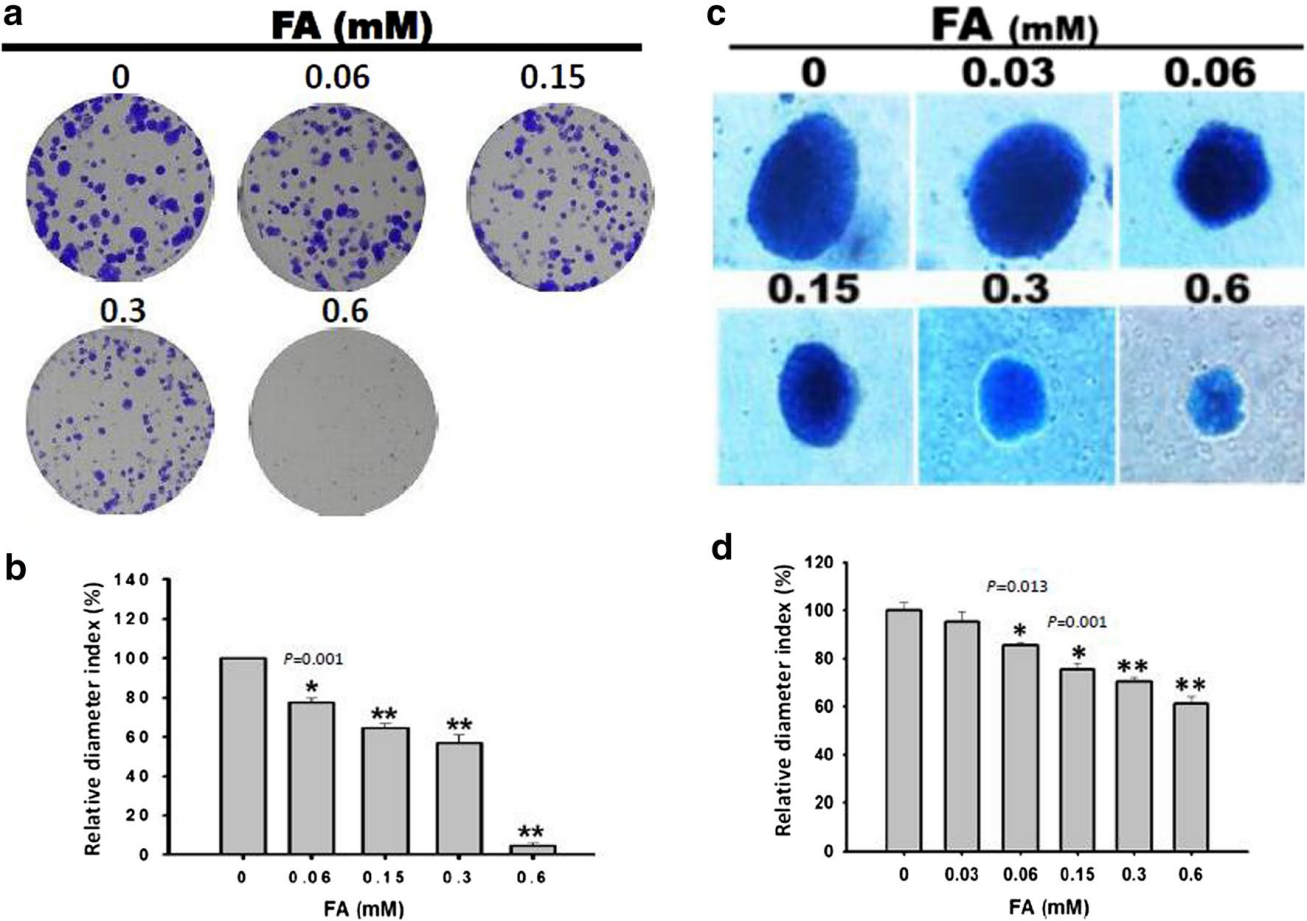
The long-term effect of *trans*-FA on colony formation and anchorage-independent growth assay of NSCLC. **a** The assessment of colony formation, a 2D indicator of cell proliferation. Adherent H1299 cells were treated with indicated concentrations for 24 h. 5 × 10 cells treated with *trans*-FA were allowed to form colonies for 11 days. Afterward, colonies were fixed and stained using crystal violet. **c** Representative results showed the formation of tumor spheres, an indicator of 3D for anchorage-independent growth. H1299 cells were treated with indicated concentrations of *trans*-FA for 13 days using the soft agar assay. **b**, **d** Quantitative analysis of results from **a** and **c** showed the number and size of tumorspheres. *P < 0.05 and **P < 0.001, respectively

As shown in Fig. 3a, *trans*-FA inhibited colony formation in H1299 cells after 11 days of treatment. The calculated colony diameters for *trans*-FA concentrations of 0, 0.06, 0.15, 0.3 and 0.6 mM were 100 ± 0.00, 77.19 ± 2.70, 64.19 ± 2.75, 56.75 ± 4.23 and 4.59 ± 1.25 % (n = 3), respectively. Figure 3c shows that long-term treatment with *trans*-FA inhibited the AIG capacity of H1299 cells. Furthermore, the results of the colony formation showed the selectively inhibitory effect of *trans*-FA on cellular proliferation between lung cancer cells H1299 and lung fibroblast cells HEL-299 (Additional file 2). The calculated colony diameters for *trans*-FA at 0, 0.03, 0.06, 0.15, 0.3 and 0.6 mM were 100 ± 3.10, 95.34 ± 4.18, 85.64 ± 1.08, 75.51 ± 2.41, 70.41 ± 1.71 and 61.36 ± 2.70 % (n = 3), respectively.

### *Trans*-FA caused moderate G_0_/G_1_ accumulation

**Fig. 4:**
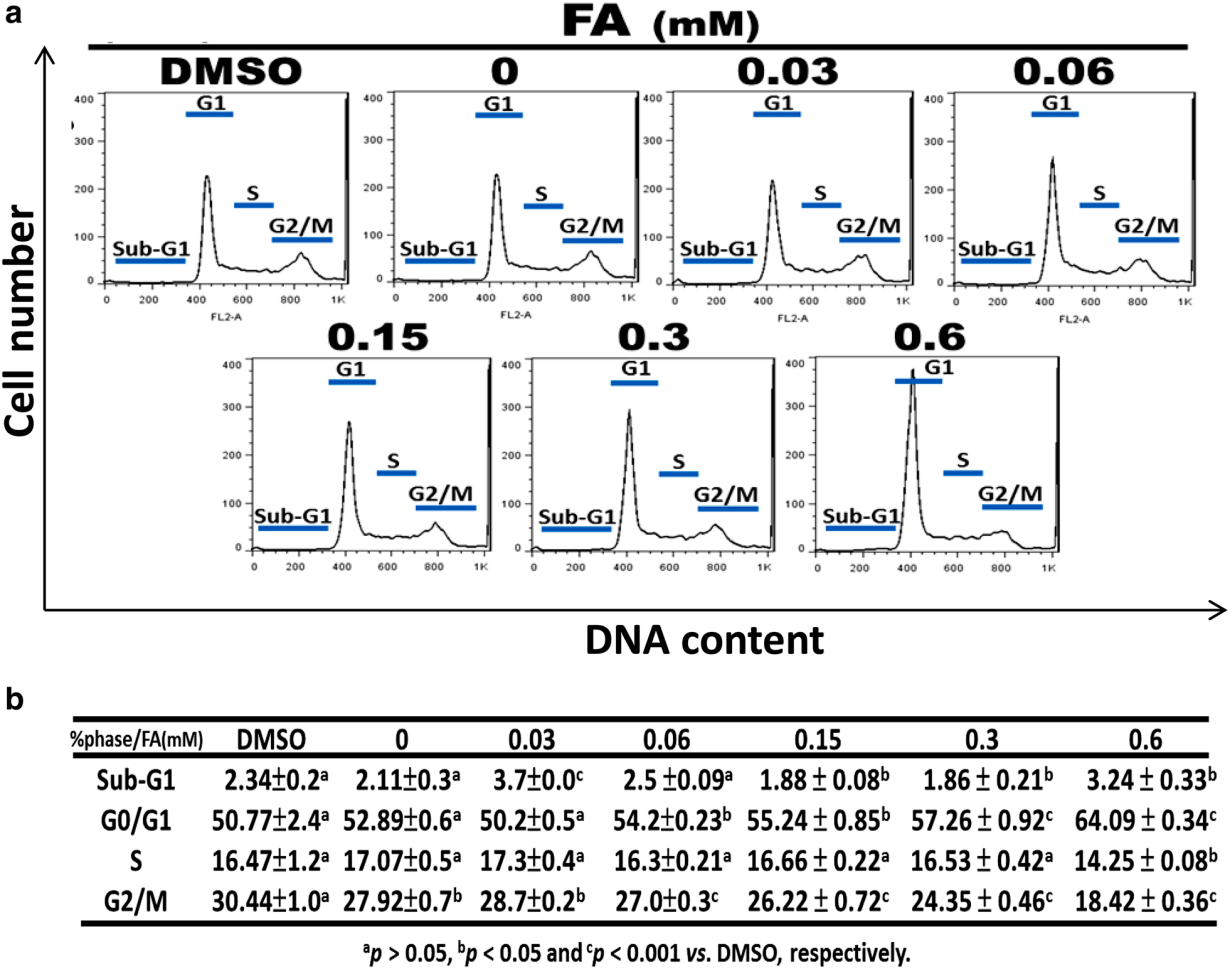
Effect of *trans*-FA on cell-cycle progression of H1299 cells. H1299 cells were treated with the indicated doses, 0.03, 0.06, 0.15, 0.3 and 0.6 mM of *trans*-FA for 48 h respectively. **a** An accumulation of G_0_/G_1_ population in *trans*-FA-treated H1299 cells and vehicle controls at 48 h. **b** The quantification analysis. Data are presented as mean ± SD (n = 3). *Different letter notations* indicate the statistical significance between control and *trans*-FA treatment groups (*a* no significance; *a* vs *b* and *a* vs *c*, statistically significant with *P* < 0.05 and 0.001, respectively)

The effects of 48 h *trans*-FA treatment on cell cycle progression in H1299 cells were examined. *Trans*-FA treatments caused the arrest of the cell cycle at G_0_/G_1_ and a decrease in the percentage of the G_2_/M phase (Fig. 4a, b).

### *Trans*-FA-induced apoptosis in lung cancer cells

**Fig. 5:**
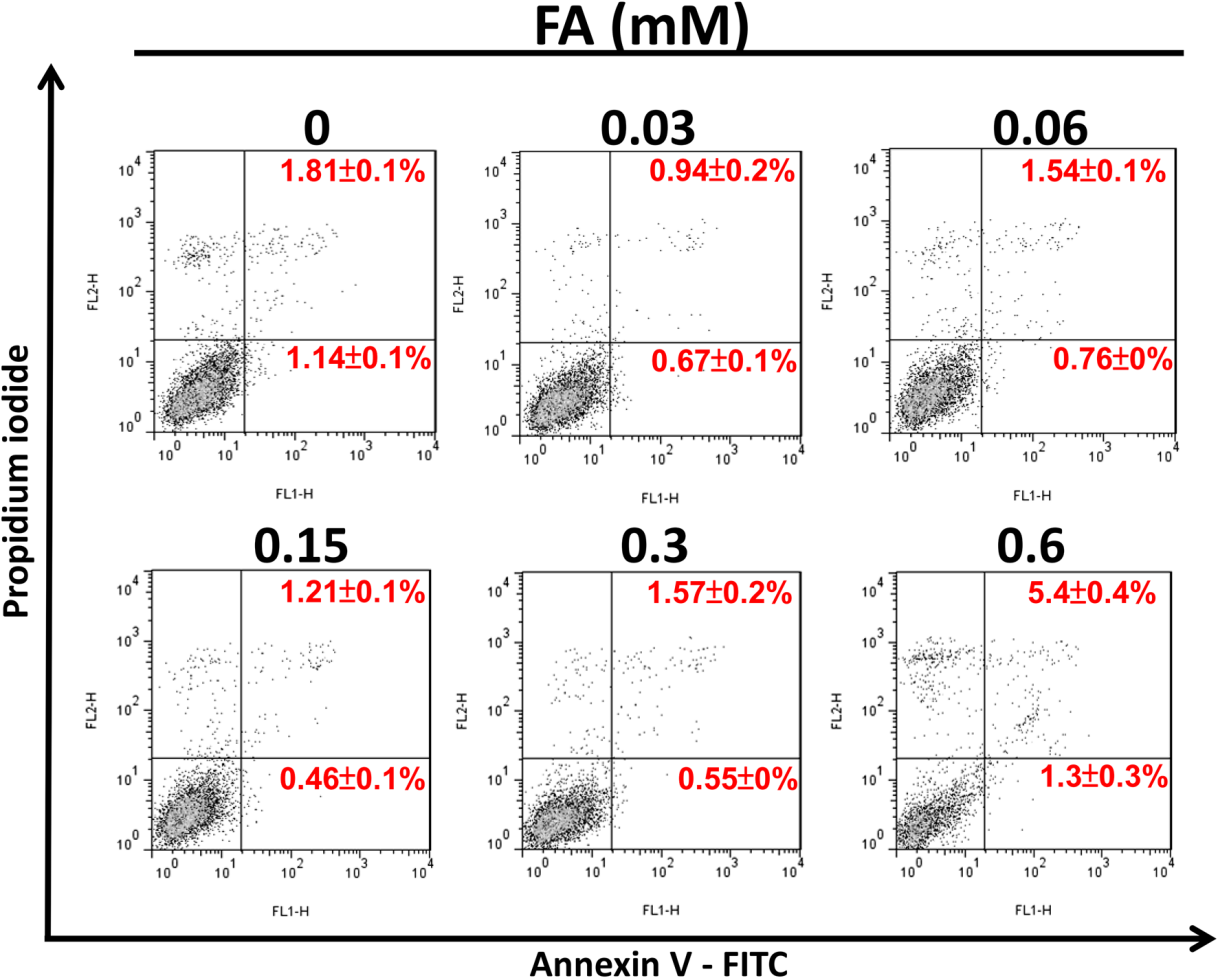
Effect of *trans*-FA on apoptosis of H1299 cells. H1299 cells were treated with indicated doses, 0.03, 0.06, 0.15, 0.3 and 0.6 mM of *trans*-FA for 48 h respectively. Annexin V/PI double staining was performed to detect the apoptosis

We investigated whether inhibition of proliferation by *trans*-FA was achieved by apoptosis in H1299 cells. Only the highest concentration (0.6 mM) of *trans*-FA increased the proportion of Annexin V^+^/PI^+^ cells (from 1.81 to 5.4 %) (Fig. 5). Other concentrations of *trans*-FA used in the study did not induce a significant increase in apoptotic populations.

### *Trans*-FA induced changes in intracellular ROS

**Fig. 6:**
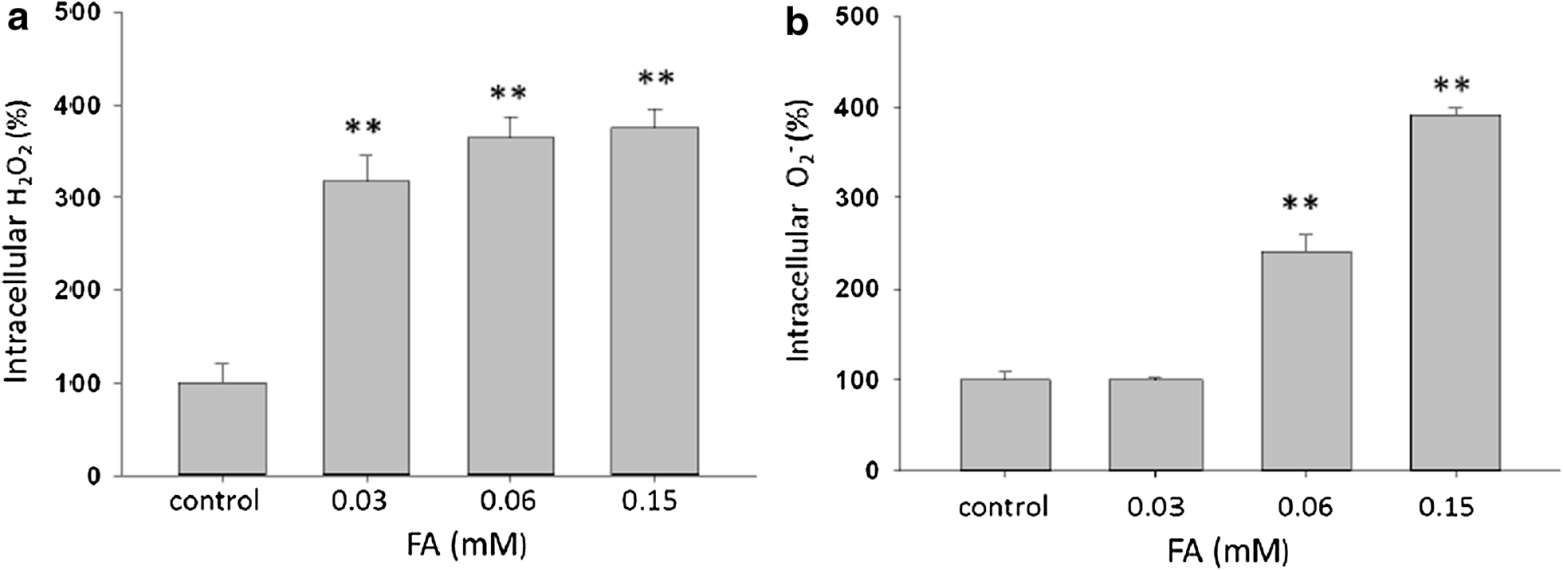
*Trans*-FA up-modulates the endogenous level of ROS in H1299 cells. 1 × 10^5^ H1299 cells were seeded onto a 6-well plate and treated with indicated doses of *trans*-FA (from 0.03 to 0.15 mM) 24 h respectively. Afterward, the induction of endogenous ROS was determined by **a** DCFDA or **b** DHE staining combined with a flow cytometry analysis. **P < 0.001 against vehicle control

The endogenous level of ROS can regulate a variety of cellular physiological processes, including survival, proliferation, angiogenesis, and signaling pathways [42]. Flow cytometry-based detection with DCFDA and DHE staining was used to detect endogenous H_2_O_2_ and O_2_^−^, respectively. As shown in Fig. 6, H_2_O_2_ concentrations of 100 ± 20, 317 ± 28, 364 ± 23 and 375 ± 20 % (n = 3) were observed in H1299 cells treated with different concentrations of *trans*-FA (0–0.15 mM) for 24 h. Furthermore, endogenous O_2_^−^ concentrations of 100 ± 9, 100 ± 2, 241 ± 19 and 392 ± 7 % (n = 3) were observed with the same *trans*-FA concentrations (Fig. 6b).

### Regulation of survival proteins by *trans*-FA

**Fig. 7:**
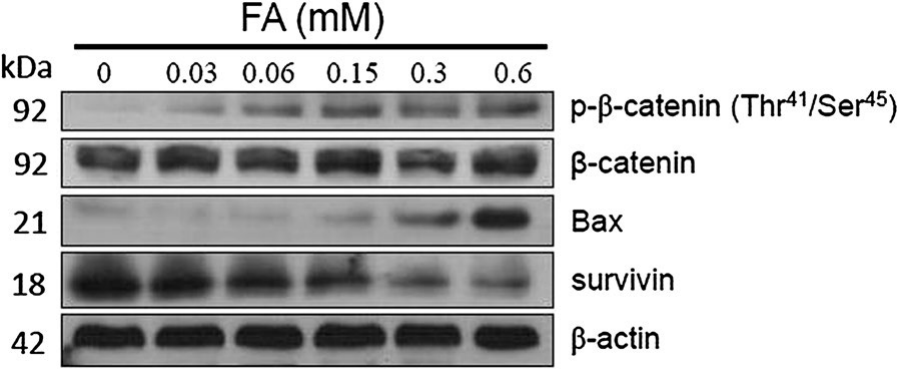
Effect of *trans*-FA on survival protein of H1299 cells. H1299 cells were treated with the indicated doses, 0.03, 0.06, 0.15, 0.3 and 0.6 mM of *trans*-FA for 48 h. Western blot assay was performed

Western blotting was used to examine whether *trans*-FA treatment affected the expression of β-catenin in H1299 cells. As shown in Fig. 7, *trans*-FA treatment increased β-catenin phosphorylation at Thr^41^/Ser^45^ but did not affect β-catenin protein levels. The anti-cancer effects of *trans*-FA might act by selective inhibition of β-catenin, a transcription factor associated with the growth and migration of H1299 cells. Anti-survival Bax protein was increased following *trans*-FA treatment in a dose-responsive manner, although the protein level of survivin was decreased.

### *Trans*-FA attenuated the motility of lung cancer cells

**Fig. 8:**
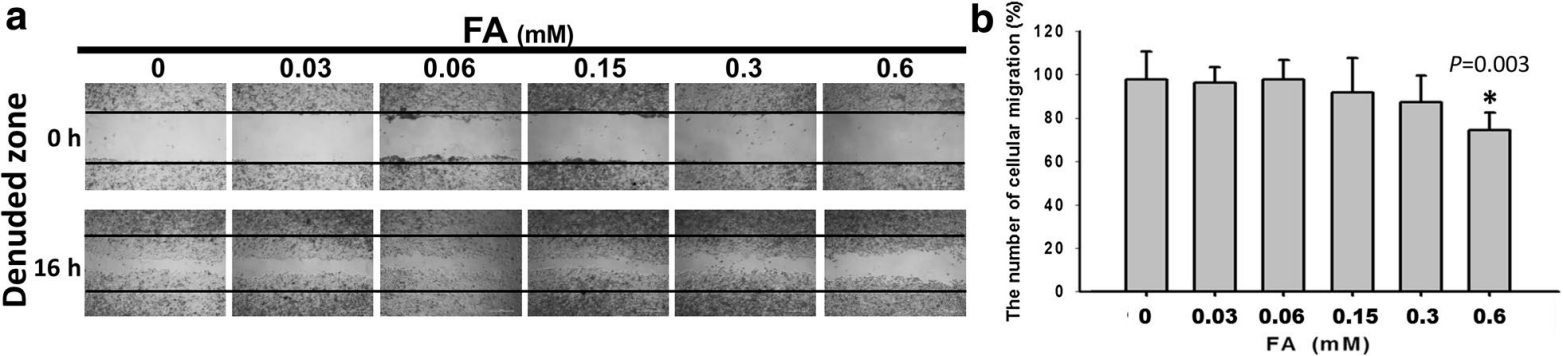
Effect of *trans*-FA on the migration of H1299 cells. *Trans*-FA inhibits cell migration of NSCLC tumor H1299 cells at the highest dose (0.6 mM). **a** 3 × 10^5^ cells were seeded in a 12-well plate and cells were scraped to create a clean 1-mm area within the confluent culture. Cells were treated with the indicated doses of 0.03, 0.06, 0.15, 0.3 and 0.6 mM of *trans*-FA for 16 h. Afterward, the wound gaps were photographed using an inverted phase-contrast microscopy. **b** The quantification analysis. *P < 0.05 for *trans*-FA treatments against vehicle control

Wound-healing assays were performed to investigate whether *trans*-FA affected migration of NSCLC cells. *trans*-FA treatment moderately attenuated the migration of H1299 lung cancer cells (Fig. 8). The area of the denuded zone was used as an index of the migratory ability of H1299 cells. The areas measured for *trans*-FA concentrations of 0, 0.03, 0.06, 0.15, 0.3 and 0.6 mM were 100 % ± 5.78, 96.86 % ± 2.69, 98.99 % ± 4.39, 93.95 % ± 6.77, 85.78 % ± 5.76 and 76.87 % ± 1.76 (n = 3), respectively.

### *Trans*-FA exerted an anti-invasion effect

**Fig. 9:**
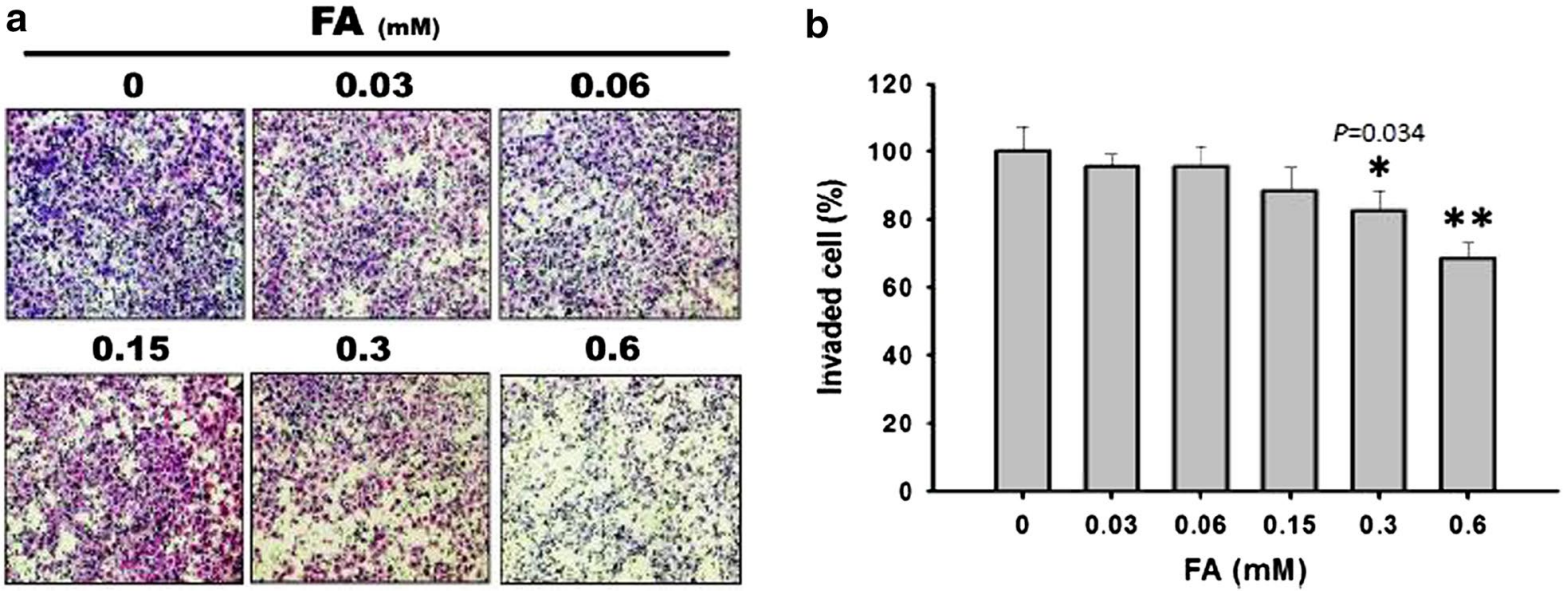
Effect of *trans*-FA on the invasion of H1299 cells. **a** Cells were treated with the indicated doses of 0.03, 0.06, 0.15, 0.3 and 0.6 mM of *trans*-FA for 16 h and stained with 0.1 % w/v Giemsa stain respectively. **b** The results of the quantificative analysis. *P < 0.05 and **P < 0.001 for *trans*-FA treatments against vehicle control, respectively

A Boyden chamber assay was used to evaluate invasion ability. After treating H1299 cells with various concentrations (from 0.03 to 0.6 mM) of *trans*-FA for 16 h, the percentages of invasive cells were 100 % ± 7.49, 95.79 % ± 3.42, 95.61 % ± 5.86, 88.39 % ± 7.01, 82.57 % ± 5.87 and 68.57 % ± 4.48 (n = 3) (Fig. 9).

### *Trans*-FA reduced the activity of MMP-2 and MMP-9

**Fig. 10:**
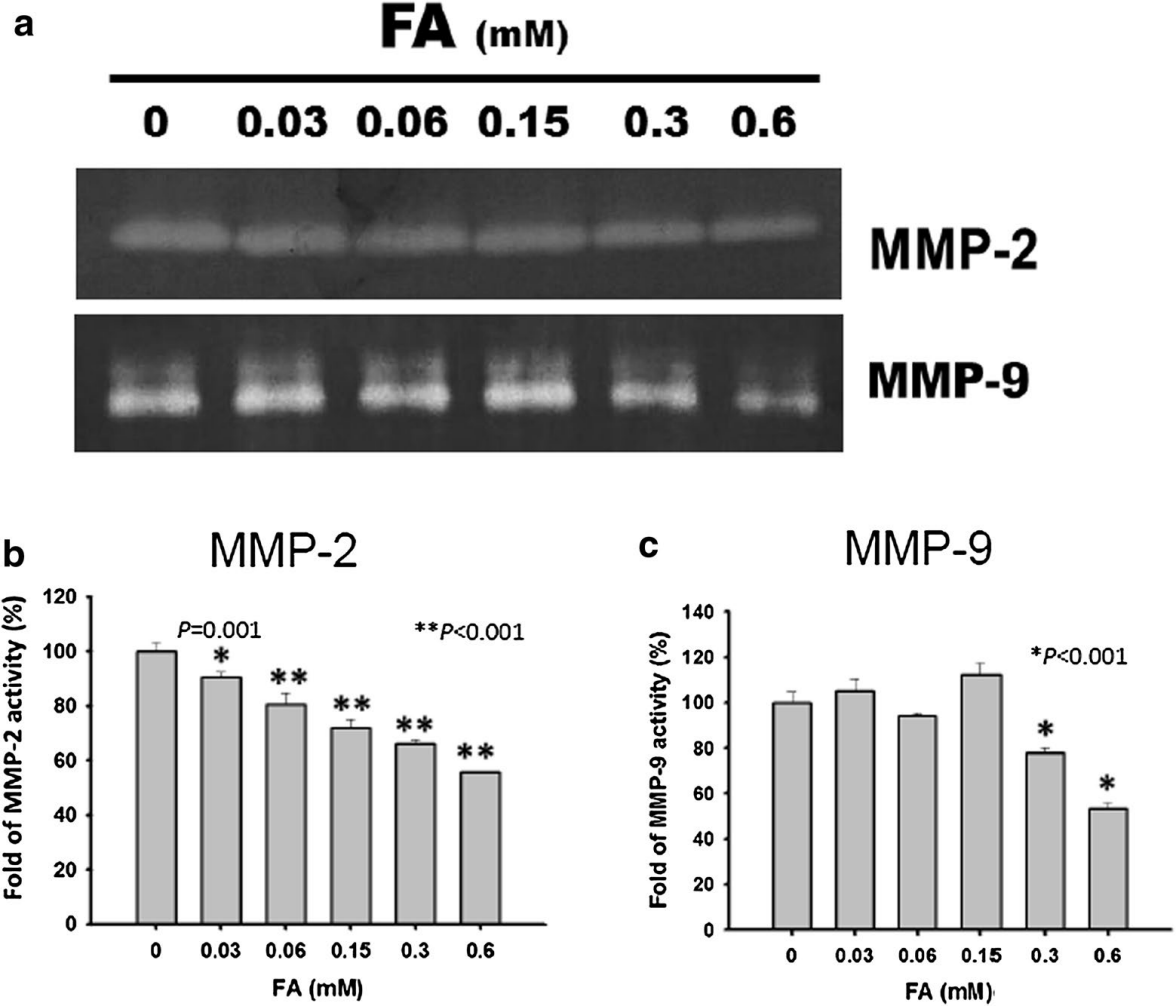
Effect of *trans*-FA on activities of MMP-2 and MMP-9. H1299 cells were treated with indicated concentrations of *trans*-FA for 24 h respectively. **a** The activities of MMP-2 and MMP-9 were determined by a gelatin zymography assay (n = 3). **b**, **c** The results of the quantificative analysis. **b** *P < 0.05 and **P < 0.001 for *trans*-FA treatments against vehicle respectively. **c** *P < 0.001 for *trans*-FA treatments against vehicle control

MMP-2 and MMP-9 are gelatinases which degrade extracellular matrix and thus regulate the ability of cells to migrate. Overexpression of MMP-2 and MMP-9 promotes cancer progression and is highly correlated with poor prognosis of cancer patients [43]. Therefore, targeting of MMP-2 and -9 represents a promising strategy for cancer treatment [44]. The activity of MMP-2 and MMP-9 was determined using a gelatin zymography assay (Fig. 10a). *Trans*-FA treatment (0, 0.03, 0.06, 0.15, 0.3 and 0.6 mM) significantly reduced the activity of MMP-2 [100 ± 2.95, 90.59 ± 1.96, 80.43 ± 4.46, 71.99 ± 2.9, 66.04 ± 1.59 and 55.87 ± 0.26 % (n = 3)] (Fig. 10b, c). The activity of MMP-9 [100 ± 4.44, 105.13 ± 5.29, 94.04 ± 0.99, 112.08 ± 5.24, 78.04 ± 1.86 and 53.32 ± 2.38 % (n = 3)] was also reduced.

## Discussion

The radical scavenger assay findings indicated the potent anti-oxidant activity of *trans*-FA (Fig. 1). Treatment with *trans*-FA for 24 or 48 h did not affect cellular morphology and proliferation of lung cancer H1299 cells (Fig. 2). However, long-term treatment with *trans*-FA attenuated colony formation and AIG, characteristics of advanced cancer, in H1299 cells (Fig. 3). *Trans*-FA induced moderate cell proliferation at the lowest concentration tested (0.03 mM). These results were consistent with previous reports that *trans*-FA promoted proliferation of MCF7 and BT20 breast cancer cells and neural progenitor cells [45, 46]. The colony formation assay also showed the discrepant inhibitory effect of *trans*-FA on cellular proliferation of lung cancer cells H1299 and lung fibroblast cells HEL-299 (Additional file 2), suggesting the potential of *trans*-FA for selectively inhibiting lung cancer. Furthermore, *trans*-FA significantly inhibited AIG capability in a dose-responsive manner.

*Trans*-FA caused an accumulation of the G_0_/G_1_ population and induced a moderate increase in the apoptotic population at the highest concentration (0.6 mM) (Fig. 4). G_0_/G_1_ cell cycle arrest is usually associated with the upregulation of cell cycle regulatory protein p15^*INK4b*^ and p21^*WAF1/Cip1*^ [47]. A recent study showed that perillyl alcohol, a natural compound purified from citrus fruits and herbs, causes G_1_ arrest and inhibits proliferation of human immortalized keratinocyte HaCaT cells through inducing p15^*INK4b*^ and p21^*WAF1/Cip1*^ [47]. Annexin V staining confirmed that the anti-cancer effects of *Trans*-FA were not mediated through apoptosis (Fig. 5).

Excess endogenous ROS may inhibit cellular growth or cause cell death [48–51]. The anti-cancer effects of *trans*-FA might correlate with increased levels of ROS in H1299 cells (Fig. 6). ROS content is higher in cancer cells than in normal cells, and ROS are reported to be involved in cancer cell migration [42]. In this study, *trans*-FA treatment caused the accumulation of both H_2_O_2_ and O2^−^. *Trans*-FA (0.03 mM) induced an increase in H_2_O_2_, but not O_2_^−^. Changes in endogenous ROS levels were assessed using the fluorescent indicators DCFDA for H_2_O_2_ and DHE for O_2_^−^ [52]. Superoxide dismutase (SOD) converts O_2_^−^ into H_2_O_2_, and is overexpressed in lung cancer compared with normal and non-malignant lung tissues [53]. Therefore, a moderate increase in O_2_^−^ might be rapidly converted into H_2_O_2_ in lung cancer cells. However, the significant increase in endogenous O_2_^−^ induced by *trans*-FA (>0.03 mM) may cause saturation of SOD capacity, preventing further conversion of O_2_^−^ to H_2_O_2_. Accordingly, increased levels of H_2_O_2_ might be the product of O_2_^−^ conversion by SOD in H1299 cells following low dose (0.03 mM) *trans*-FA treatment.

β-catenin is a transcription factor involved in cell growth and cell migration pathways. Wnt/β-catenin signaling is thus essential for the maintenance of neuronal progenitor proliferation [54]. However, phosphorylated β-catenin is inactivated and undergoes proteasomal degradation, causing the inhibition of cell growth [55].

With respect to tumorigenesis, constitutive activation or overexpression of β-catenin is frequently observed in cancers, including rectal cancer [56], colon cancer [57], breast cancer [58], prostate cancer [59], glioma [60], and lung cancer [61]. Furthermore, overexpression of β-catenin enhances the expression of cyclin D1, a critical factor for G_1_/S transition during cell cycle progression in colon carcinoma cells [62]. *S*-adenosylmethionine and its metabolite, methylthioadenosine, inhibited β-catenin signaling by multiple mechanisms in colon cancer, and thus might have the potential to prevent tumorigenesis [63]. Furthermore, Wnt/β-catenin signaling was shown to be a potent activator of ROS generation, resulting in DNA damage and acceleration of cellular senescence [64]. Furthermore, Wnt/β-catenin signaling potently activated ROS generation in mesenchymal stem cells [64–66].

To clarify the underlying mechanism of *trans*-FA-induced anti-lung cancer activities, we examined whether *trans*-FA could affect the expression of cell proliferation-related transcription factor β-catenin using western blotting (Fig. 7). Our results demonstrated that *trans*-FA treatment promoted the phosphorylation of β-catenin at residues Thr^41^ and Ser^45^ [55] and led to the proteasomal degradation of cytoplasmic β-catenin, causing the downregulation of β-catenin protein levels. The Wnt pathway regulated MMP-2/-9 expression by directly targeting the MMP promoter through T-cell factor (TCF), a β-catenin interacting partner, therefore promoting cellular migration [67]. In effector T cells, endothelial cell-derived Wnt induced the expression of MMP-2/-9 through activating the Frizzled receptors to regulate the transmigration of T cells. In contrast, Wnt signaling blockade reduced the migration of effector T cells in vitro [67].

In addition to β-catenin, we also examined the role of pro-survival protein Bax, a key anti-survival factor, can promote apoptosis by binding to and antagonizing pro-survival Bcl-2 proteins such as Bcl-2 or Bcl-xL [68]. Conversely, survivin is a member of the inhibitor of apoptosis (IAP) family and acts as an inhibitor of caspase activation, thereby negatively regulating apoptosis or programmed cell death [69]. Both the Bcl-2 family and IAP proteins are critical regulators of cell proliferation and survival. In our study, the significant changes in Bax and survivin expression occurred alongside the anti-proliferation effects observed following *trans*-FA treatment (Fig. 7). As shown using colony formation and AIG assays, *trans*-FA treatment might impair cell proliferation of H1299 cells. Apart from in cells treated with higher concentrations (0.3 and 0.6 mM) of *trans*-FA, no significant increase in the population of apoptotic cells was detected. Survivin is considered an apoptosis inhibitor which promotes cellular proliferation, although decreased expression of survivin may not always cause apoptosis [69]. Ito et al. showed that both human hepatocellular carcinoma (HCC) cell lines and patient tissues expressed high levels of survivin mRNA, with detectable levels not found in normal and non-tumor areas of liver [70]. Survivin expression may be an indicator of cellular proliferation but not apoptosis in HCC tissues [70]. The degradation or expression Bax may represent a threshold for inducing apoptosis [71]. These results might explain how *trans*-FA treatment caused an increase in Bax protein expression but did not significantly induce apoptosis in H1299 cells at most concentrations tested (0.015–0.15 mM). These observations suggest that *trans*-FA treatment attenuates cellular proliferation rather than cellular survival. Therefore, the results of the proliferation assay imply that the anti-migratory effect of *trans*-FA may also be mediated by regulating the balance of pro-survival and anti-survival proteins in lung cancer cells.

Extracellular matrix-degrading MMPs, especially MMP-2 and MMP-9, are involved in the metastasis of cancer cells [72]. *Trans*-FA treatment inhibited the migration and invasion of lung cancer cells and concurrently attenuated the activities of both MMP-2 and MMP-9 (Figs. 8, 9, 10). These observations suggest a positive correlation between MMP activity and *trans*-FA-induced anti-migration in lung cancer cells.

**Fig. 11:**
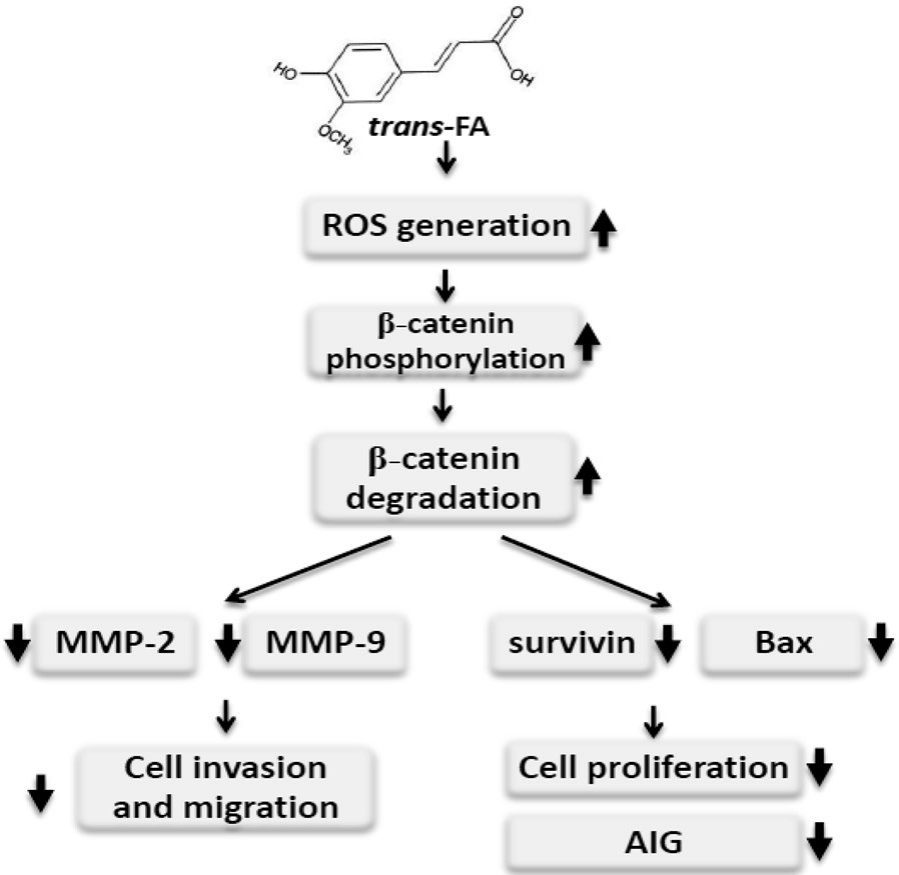
Schematic diagram of hypothesized mechanism of *trans*-FA effect of lung cancer cells. *Trans*-FA induced ROS leading to degradation of phosphorylated β-catenin. *Trans*-FA induced regulation of pro-survival proteins survivin and anti-survival protein Bax caused the anti-proliferation of lung cancer H1299 cells. *Trans*-FA also reduced the activity of both MMP-2 and MMP-9, causing the down-regulation of migration of H1299 cells

Based on these observations, we propose that the anti-lung cancer effects of *trans*-FA might act through the modulation of endogenous ROS and β-catenin stabilization. *Trans*-FA induced the production of endogenous ROS and may cause β-catenin phosphorylation, resulting in proteasomal degradation. In addition, *trans*-FA regulated the balance between pro-survival and pro-apoptosis signals and downregulated the activities of metastasis-associated MMP-2 and MMP-9 (Fig. 11).

## Conclusion

Various concentrations (0.06–0.6 mM) of *trans*-FA exert both anti-proliferation and anti-migration effects in the human lung cancer cell line H1299.

## Additional files

10.1186/s13020-016-0116-7 DPPH radical-scavenging capacity of vitamin C as a positive control. (A) Vitamin C as a positive control in DPPH assay. (B) Quantificative analysis of (A). The radical-scavenging capacity of Vitamin C at indicated concentrations was quantified as the percentage decrease in absorbance at 492 nm against the blank control. **P* < 0.05 and ***P* < 0.001 for *trans-*FA treatments against vehicle respectively.
10.1186/s13020-016-0116-7 Discrepant proliferative effect of trans-FA on long-term expansion of lung cancer cells and lung fibroblast. Lung fibroblast HEL-299 and NSCLC tumor cells H1299 were treated with indicated concentrations of trans-FA respectively. Afterward, cells were fixed with glutaraldehyde and stained with crystal violet.

## Abbreviations

AAPH: 2,2′-azobis-2-amidinopropane dihydrochloride
AIG: anchorage-independent growth
AMPK: AMP activated protein kinase
Bax: Bcl-2-associated X protein
Bcl-2: B-cell lymphoma 2
Bcl-xL: B-cell lymphoma-extra large
DCFDA: 2′,7′-dichlorofluorescin diacetate
DHE: dihydroethidium
DMSO: dimethyl sulphoxide
DPPH: 2,2-diphenyl-1-picrylhydrazyl
ECL: enhanced chemiluminescence
FA: ferulic acid
NSCLC: non-small cell lung cancer
IAP: inhibitor of apoptosis
MMP: matrix metallopeptidase
mTOR: mammalian target of rapamycin
PBS: phosphate-buffered saline
PI: propidium iodide
RNase A: ribonuclease A
ROS: reactive oxygen species
SD: standard deviation
SDS: sodium dodecyl sulfate
SOD: superoxide dismutase
TCF: T cell factor
