# Supplementary figures and images for: Inhibitory effect of trans-ferulic acid on proliferation and migration of human lung cancer cells accompanied with increased endogenous reactive oxygen species and β-catenin instability
Source: Chin Med. 2016 Oct 1;11:45. doi: 10.1186/s13020-016-0116-7 (PMC5045596; doi:10.1186/s13020-016-0116-7)

Additional file 1.

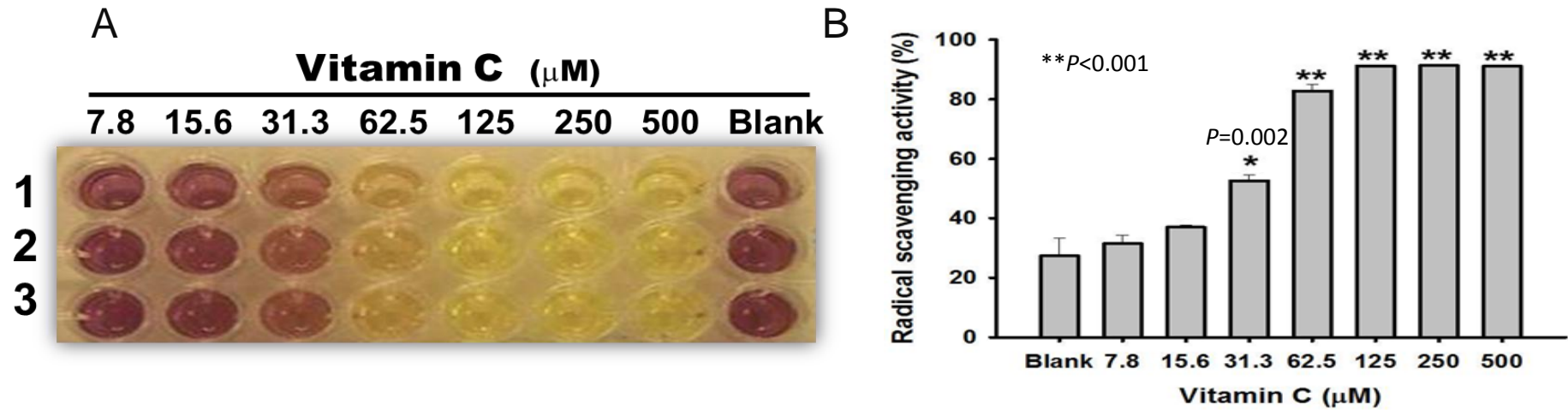

Supplement: Supplementary file 1 — 10.1186/s13020-016-0116-7 DPPH radical-scavenging capacity of vitamin C as a positive control. (A) Vitamin C as a positive control in DPPH assay. (B) Quantificative analysis of (A). The radical-scavenging capacity of Vitamin C at indicated concentrations was quantified as the percentage decrease in absorbance at 492 nm against the blank control. *P < 0.05 and **P < 0.001 for trans-FA treatments against vehicle respectively. [file 13020_2016_116_MOESM1_ESM.pdf]

Additional file 2.

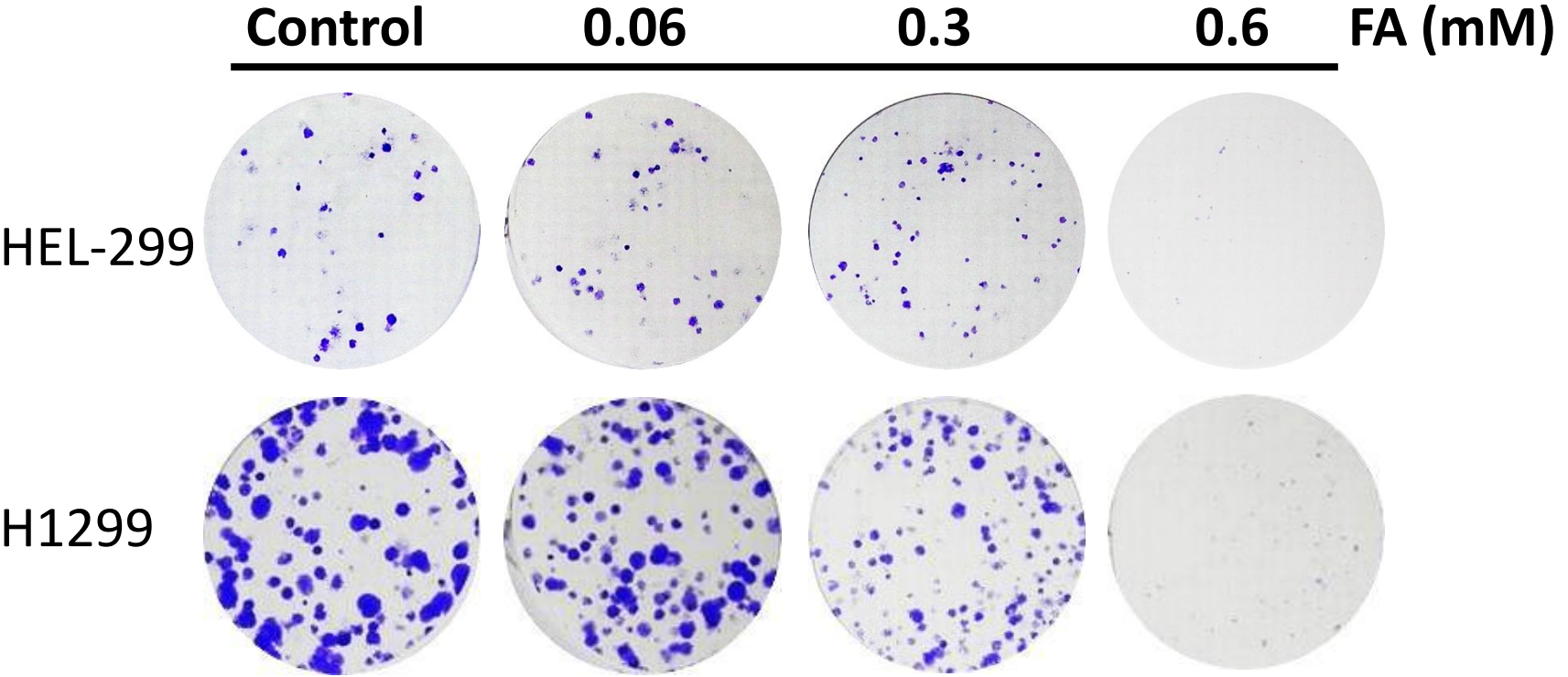

Supplement: Supplementary file 2 — 10.1186/s13020-016-0116-7 Discrepant proliferative effect of trans-FA on long-term expansion of lung cancer cells and lung fibroblast. Lung fibroblast HEL-299 and NSCLC tumor cells H1299 were treated with indicated concentrations of trans-FA respectively. Afterward, cells were fixed with glutaraldehyde and stained with crystal violet. [file 13020_2016_116_MOESM2_ESM.pdf]
